# Therapeutic Hypothermia in Stroke

**DOI:** 10.4061/2011/157969

**Published:** 2012-01-24

**Authors:** Midori A. Yenari, Fred Colbourne, Thomas M. Hemmen, Hyung Soo Han, Derk Krieger

**Affiliations:** ^1^Department of Neurology, University of California, San Francisco Neurology (127) VAMC 4150 Clement Street, San Francisco, CA 94121, USA; ^2^Department of Psychology, University of Alberta, Edmonton, AB, Canada T6G 2E9; ^3^Department of Neurosciences, University of California, San Diego, 9444 Campus Point Drive, La Jolla, CA 92037, USA; ^4^Department of Physiology, Kyungpook National University School of Medicine, Daegu 700-422, Republic of Korea; ^5^Departments of Neurology, University of Copenhagen, Denmark

For nearly 10 years, therapeutic mild hypothermia has become increasingly recognized to positively influence neurological outcome in humans following acute brain injuries, namely, ischemic brain injury due to cardiac arrest and hypoxic-ischemic encephalopathy in neonates. In the laboratory, hypothermia is perhaps the most robust and consistent neuroprotectant studied to date. It has been shown to both suppress and enhance many factors leading to ultimate tissue preservation and better outcome. Now that it has been shown to be effective in humans, this special issue surveys the various directions this area of research is taking, from using it as a model of neuroprotection in the laboratory to refining the technique and exploring ways of applying hypothermia to other neurological conditions, especially stroke. 

Y. Shintani et al. provide an overview of how state-of-the-art molecular gene and protein profiling technologies can be applied to models of hypothermic neuroprotection to reveal potentially new signaling pathways and to identify potential therapeutic targets. This strategy was then demonstrated by H. S. Han et al. who discovered that hypothermia upregulates extracellular signal regulated kinase (ERK-1/2) and that ERK-1/2 upregulation might be responsible for the anti-inflammatory properties of cooling.

Therapeutic hypothermia has become increasingly embraced by the medical community as a means of improving neurological outcome in certain conditions. However, this intervention still has its limitations. Since these trials were published, centers have struggled to determine optimal cooling techniques, such as surface cooling versus endovascular methods. A. F. Caulfield et al. report their experience with both approaches at an academic stroke center. Another critical question is whether therapeutic cooling can be offered in community settings or whether patients need to be referred to tertiary centers. The study by M. P. Shah and colleagues show that it is possible to implement a cooling protocol in a community hospital, provided that there is a team approach in place, involving relevant medical services.

Encouraging clinical studies in cardiac arrest and hypoxic-ischemic encephalopathy patients has fueled further interest in pursuing therapeutic cooling in stroke victims. However, stroke poses additional challenges since most stroke victims are elderly with many comorbidities. Stroke patients are also generally awake, making cooling more difficult due to the problem of shivering. Nevertheless, preclinical studies continue to refine optimal parameters for such an intervention in stroke models, including defining the limits of hypothermic protection, whether cooling might be combined with other therapies and understanding where preclinical models might fall short of the clinical condition. These issues are explored in reviews by H. G. Zhao and Steinberg and Zgavc et al. Still, there is an urgent need for larger, prospective studies to determine if hypothermia could be used in stroke patients. Bench scientists, translational researchers, and clinicians alike must work together towards this goal.

*Midori A. Yenari* *Midori A. Yenari* *Fred Colbourne* *Fred Colbourne* *Thomas M. Hemmen* *Thomas M. Hemmen* *Hyung Soo Han* *Hyung Soo Han* *Derk Krieger* *Derk Krieger*

